# A data extraction template for the behaviour change intervention ontology

**DOI:** 10.12688/wellcomeopenres.20872.1

**Published:** 2024-03-26

**Authors:** Emma Norris, Lisa Zhang, Kelsey Wuerstl, Hannah Froome, Susan Michie

**Affiliations:** 1 Department of Health Sciences, Brunel University London, London, England, UK; 2 Centre for Behaviour Change, University College London, London, England, UK; 3 School of Health & Exercise Sciences, The University of British Columbia, Vancouver, British Columbia, Canada

**Keywords:** evidence synthesis, data extraction, annotation, ontology, behaviour change intervention, intervention reporting

## Abstract

**Background:**

The Behaviour Change Intervention Ontology (BCIO) aims to improve the clarity, completeness and consistency of reporting within intervention descriptions and evidence synthesis. However, a recommended method for transparently annotating intervention evaluation reports using the BCIO does not currently exist. This study aimed to develop a data extraction template for annotating using the BCIO.

**Methods:**

The BCIO data extraction template was developed in four stages: i) scoping review of papers citing component ontologies within the BCIO, ii) development of a draft template, iii) piloting and revising the template, and iv) dissemination and maintenance of the template.

**Results:**

A prototype data extraction template using Microsoft Excel was developed based on BCIO annotations from 14 papers. The ‘BCIO data extraction template v1’ was produced following piloting and revision, incorporating a facility for user feedback.

**Discussion:**

This data extraction template provides a single, accessible resource to extract all necessary characteristics of behaviour change intervention scenarios. It can be used to annotate the presence of BCIO entities for evidence synthesis, including systematic reviews. In the future, we will update this template based on feedback from the community, additions of newly published ontologies within the BCIO, and revisions to existing ontologies.

## Plain language summary

Behaviour change interventions are often reported in an inconsistent and incomplete manner in study reports. This makes it difficult to build knowledge and predict outcomes. There is a need for a shared language to describe behaviour change interventions. This need was met using ‘ontologies’, which are classification systems that represent knowledge in a standardised way. The Behaviour Change Intervention Ontology (BCIO) has been developed to describe the different aspects of interventions in a way that is precise enough for computers as well as humans to ‘read’ study findings. The BCIO can be used to extract information from study reports for evidence synthesis, such as systematic literature reviews. To meet the need for a resource for annotating (coding) study reports according to the BCIO, we developed a data extraction template. The template was developed in four stages: i) reviewing existing papers using the BCIO, ii) development of a draft template, iii) piloting and revising the template, and iv) dissemination and maintenance of the template. The resulting resource is an accessible, easy-to-use template to assist with specifying the content of published papers reporting interventions and their evaluation. The template will be updated based on user feedback and future revisions to the BCIO.

## Introduction

Behaviour change interventions vary considerably in their characteristics including their content and delivery, context (setting and population), target behaviours, and mechanisms of action ( Michie & Johnston, 2017). However, interventions are often poorly reported, with inconsistent or ambiguous use of scientific terminology ( Ioannidis *et al.*, 2014; Michie *et al.*, 2009). This makes it difficult to replicate and scale up interventions, and to synthesise evidence to build knowledge ( Wright *et al.*, 2020). Although reporting guidelines (e.g., Hoffmann *et al.*, 2014; Montgomery *et al.*, 2018) have improved the clarity and completeness of reporting, there is a need for a common, shared vocabulary to standardise the classification of key aspects of behaviour change interventions.

****Ontologies**** can meet this need by integrating knowledge across different disciplines, domains and data types (see Appendix 1 for glossary of terms that are bolded and italicised). Ontologies are formal classification systems for representing the world in terms of ****classes**** of ****entities**** (anything that exists in the universe) and ****relations**** between entities ( Arp *et al.*, 2015). Every entity consists of a label and a formal, unambiguous definition which are specified using logic-based language and unique identifiers, allowing it to be computer-readable ( Hastings, 2017). In several scientific fields, ontologies have helped create a common language and a ‘controlled vocabulary’ (standardised sets of terms) to organise and represent knowledge ( Hastings, 2017; Sharp *et al.*, 2023; Smith *et al.*, 2007). The ****Open Biological and Biomedical Ontology (OBO) Foundry**** hosts a collection of coordinated, ****interoperable**** ontologies ( Smith *et al.*, 2007). They follow principles, such as being openly available (see https://obofoundry.org/principles/fp-000-summary.html). The precision of ontologies means that ontologies can support the application of artificial intelligence and machine learning approaches in data extraction, evidence synthesis, and outcome prediction ( Hastings *et al.*, 2023; West *et al.*, 2023a). This can reduce research waste and maximise the speed and scale of evidence accumulation.

The Human Behaviour-Change Project ( Michie *et al.*, 2017) has developed a Behaviour Change Intervention Ontology (BCIO) as part of constructing an automated ‘knowledge system’ for gathering information from reports of behaviour change intervention evaluations, and using this information to predict intervention outcomes in novel scenarios ( Michie *et al.*, 2021). The BCIO contains more than 1000 entities relating to intervention content (BCTs; Marques *et al.*, 2023), delivery (including mode ( Marques *et al.*, 2020), source ( Norris *et al.*, 2021), style ( Wright *et al.*, 2023) and schedule), engagement, target population, setting ( Norris *et al.*, 2020), target behaviour, and mechanisms of action ( Schenk *et al.*, 2023). It follows the OBO foundry principles for good practice in ontology development. The upper level BCIO is described in Michie *et al.* (2021), and the method for developing the lower level (component) ontologies is described in Wright *et al.* (2020).

The BCIO can be used to enable writing clear and comprehensive study protocols and study reports by using its entity labels and definitions. It can also be used to synthesise evidence as it clarifies what is the same, and what is different, across studies. Because entities each have a unique identifier, information represented by ontologies can be used in artificial intelligence and machine learning to predict behavioural outcomes.

This article focuses on the development of a data extraction template to assist in evidence synthesis through the annotation of study reports. Existing classification frameworks in behavioural science, such as the Behaviour Change Technique Taxonomy v1 (BCTTv1; Michie *et al.*, 2013), have been an important and reliable method for annotating the content of interventions, as they provide shared terminology and structures for conceptualising behaviour change interventions. The BCIO is an advance as it allows all aspects of intervention scenarios (e.g. content, delivery, context, engagement, mechanisms of action) to be annotated in a systematic and standardized way. Sheils *et al.* (2024) identified a need for “materials in how to code according to this revised framework”, highlighting that an accessible resource is necessary to support researchers and practitioners to apply the BCIO in evidence synthesis. For example, in annotating BCIO entities for a systematic review of digital interventions to address children’s fruit and vegetable consumption ( Froome *et al.*, 2023), it was apparent that there was a need for a single data extraction template covering all component ontologies within the BCIO. It can also serve as a key entry point for using the BCIO more broadly for other purposes, such as intervention development.

Specialist software such as EPPI-Reviewer, Covidence, and RevMan are excellent tools to support data extraction. However, researchers would need to create data extraction sheets by manually inputting entities from the BCIO into the software system. Given the large number of entities, this is inefficient and creates an unnecessary burden on researchers. Here we report the development of an accessible and standardised data extraction template, containing every BCIO entity label, definition and ****Uniform Resource Identifier (URI)**** within its hierarchical structure.

### Aim

This study aimed to develop a data extraction template for annotating intervention evaluation reports according to the BCIO.

## Methods

### Ethical statement

Ethical approval was granted by the University College London’s ethics committee (CEHP/2020/579).

### i) Scoping review of papers citing the BCIO

Forward citation searching was performed in November 2023 on published papers reporting the development of lower-level ontologies within the BCIO, namely Mode of delivery ( Marques *et al.*, 2020), Intervention Setting ( Norris *et al.*, 2020), Intervention Source ( Norris *et al.*, 2021), Mechanisms of Action ( Schenk *et al.*, 2023), Behaviour Change Technique ( Marques *et al.*, 2023) and Style of delivery ( Wright *et al.*, 2023) ontologies ( Papaioannou *et al.*, 2010). Google Scholar was used for forward citation searching, as it is widely viewed as the most comprehensive citation tool ( Martín-Martín *et al.*, 2021), including citations from grey literature and preprints (e.g on PsyArXiV, MetaArXiv). Information on papers citing and using each ontology was extracted onto a Microsoft Excel template ( Table 1). It included rows for each lower-level ontology and columns for: a) number of papers citing the ontology, b) in-text reporting of annotation using the ontology: number of papers, references and quotes, c) supporting documents featuring annotation using the ontology: number of papers, references, nature of reporting within the supporting documents and links to these documents. Data extraction from all papers citing the ontologies was split between two authors (EN & LZ).

**Table 1.** Forward citation searching for BCIO data extraction on Google Scholar.

Ontology	Number of papers citing ontology	Number of papers with in-text reported BCIO coding	Reference for papers with in-text reported BCIO coding	Example quotes of reported BCIO coding (methods & results)	Number of papers with supporting documents with BCIO coding	Reference for papers with supporting documents with BCIO coding	Nature of these supporting documents	Links for these documents (if available)
**Mode of delivery**	57	13	Sys Review: Jackson, S., Brown, J., Norris, E., Livingstone-Banks, J., Hayes, E., & Lindson, N. (2022). Mindfulness for smoking cessation. *Cochrane Database of Systematic Reviews*, (4).	Text within Characteristics of Included studies table: e.g "Mode of delivery: face-to-face (group), smartphone app.."	4	Sys Review protocol: Silva, C., Presseau, J., Dinsmore, J., van Allen, Z., & Marques, M. (2022). Protocol for two interrelated systematic reviews of multiple health behaviour change interventions in healthcare. PsyArXiv. https://psyarxiv.com/7dwrv/download?format=pdf	Upper levels only	https://osf.io/3cbmk
			Interv development: Umaefulam, V., Wilson, M., Boucher, M. C., Brent, M. H., Dogba, M. J., Drescher, O., ... & Presseau, J. (2023). The co-development of a linguistic and culturally tailored tele-retinopathy screening intervention for immigrants living with diabetes from China and African-Caribbean countries in Ottawa, Canada. BMC Health Services Research, 23(1), 1–19.	Method: "We identified the modes and settings of delivering behaviour change interventions [37, 38], agreed on materials to create, prototypes, and how to integrate other barriers and effective strategies not identified in the co-development workshops".		Interv development: Umaefulam, V., Wilson, M., Boucher, M. C., Brent, M. H., Dogba, M. J., Drescher, O., ... & Presseau, J. (2023). The co-development of a linguistic and culturally tailored tele-retinopathy screening intervention for immigrants living with diabetes from China and African-Caribbean countries in Ottawa, Canada. BMC Health Services Research, 23(1), 1–19.	Coding of intervention activities according to ontology 0 Additional file 4 e.g "Face-to-face and at-a-distance"	https://static-content.springer.com/esm/art%3A10.1186%2Fs12913-023-09329-3/MediaObjects/12913_2023_9329_MOESM4_ESM.xlsx
			Scoping review: Wuerstl, K. R., Todd, K., Lawrason, S., Shwed, A., Holmes, B., & Gainforth, H. L. (2023). Theoretical components of smoking cessation interventions for persons with physical disabilities: A scoping review. Addictive Behaviors, 107762.	Results: "A total of 113 modes of delivery (comprised of 22 unique modes of delivery) were extracted from the included articles. There were 86 modes of delivery extracted from the intervention arms and 27 from the control arms. Interventions reported an average of 12.5 modes of delivery, ranging from five to fourteen..."		Scoping review: Encantado, J., Palmeira, A. L., Silva, C., Sniehotta, F. F., Stubbs, R. J., Gouveia, M. J., ... & Marques, M. M. (2022). What goes on in digital behaviour change interventions for weight loss maintenance targeting physical activity: A scoping review. Digital Health, 8, 20552076221129089.	Names and numbers of papers coded within each ontology entity	https://journals.sagepub.com/doi/suppl/10.1177/20552076221129089/suppl_file/sj-xlsx-3-dhj-10.1177_20552076221129089.xlsx
			Intervention: McMahon, S. K., Macheledt, K., Choma, E. A., Lewis, B. A., Guan, W., Wyman, J. F., & Rothman, A. J. (2023). Rethinking how and when to report descriptions of behavior change content within interventions: a case study of an ongoing physical activity trial (ready steady 3.0). Translational Behavioral Medicine, 13(6), 368–379.	Method: "The informational mode of delivery was primarily face-to-face via small group meetings augmented with printed materials in a workbook and electronic materials in the form of a wearable activity monitor (e.g., step count, physical activity minutes, distance)."		Scoping review: Wuerstl, K. R., Todd, K., Lawrason, S., Shwed, A., Holmes, B., & Gainforth, H. L. (2023). Theoretical components of smoking cessation interventions for persons with physical disabilities: A scoping review. Addictive Behaviors, 107762.	Full ontology	https://osf.io/k5pe4
			Scoping review: Encantado, J., Palmeira, A. L., Silva, C., Sniehotta, F. F., Stubbs, R. J., Gouveia, M. J., ... & Marques, M. M. (2022). What goes on in digital behaviour change interventions for weight loss maintenance targeting physical activity: A scoping review. Digital Health, 8, 20552076221129089.	Results: "Only one study was entirely digital and automated. The other 10 studies also included non-automatic distant human interaction and four of these reported additional face-to-face interaction for delivering intervention content..."				
			Interv development: Giroux, E.E., Casemore, S., Clarke, T.Y. et al. Enhancing participation while aging with spinal cord injury: applying behaviour change frameworks to develop intervention recommendations. Spinal Cord 59, 665–674 (2021). https://doi.org/10.1038/s41393-020-00555-8	Results: "Against the MoDTv0, digital (e.g., websites, e-mail, television commercials, social media) was the most commonly coded category. Online videos, although not a MoDTv0 category, were also identified..."				
			Interv development: Osborne, C. & Norris, E. (2022) Pre-registration as behaviour: developing an evidence-based intervention specification to increase pre-registration uptake by researchers using the Behaviour Change Wheel, Cogent Psychology, 9:1, DOI: 10.1080/23311908.2022.2066304	Results: "..face to face (BCIO:011003); at a distance (BCIO:011004); email (BCIO:011025); and website (BCIO:011027) were selected."				
			Interv development: McClatchey, K, Sheldon, A, Steed, L, et al. Development of theoretically informed audit and feedback: an exemplar from a complex implementation strategy to improve asthma self-management in UK primary care. J Eval Clin Pract. 2023; 1–15. doi: 10.1111/jep.13895	"This aligns to the following upper‐level classes of the Mode of Delivery Ontology 16: informational mode of delivery, group‐based mode of delivery, asynchronous mode of delivery and push mode of delivery…"				
			Interv development: Shwed A, Giroux EE, Hoekstra F, et al. Supporting meaningful research partnerships: an interview study applying behavior change theory to develop relevant recommendations for researchers. Transl Behav Med. 2023;13(11):833–844. doi: 10.1093/tbm/ibad040	Results: "...When discussing the delivery of support tools and resources related to the IKT Guiding Principles, modes of delivery involved information delivery (n = 17, 100%) from human interaction (n = 5, 29%) at a distance (n = 5, 29%); printed material (n = 3, 18%) through printed publications (n = 3, 18%); electronic (n = 17, 100%) via the computer (n = 2, 12%) and calls (n = 2, 12%), specifically video calls (n = 4, 24%)..."				
			Interv development: Keller MS, Carrascoza-Bolanos J, Breda K, et alIdentifying barriers and facilitators to deprescribing benzodiazepines and sedative hypnotics in the hospital setting using the Theoretical Domains Framework and the Capability, Opportunity, Motivation and Behaviour (COM-B) Model: a qualitative studyBMJ Open 2023;13:e066234. doi: 10.1136/bmjopen-2022-066234	Method: "..the mode of delivery to implement the Behaviour Change Technique of demonstrating the new process to other surgeons might be delivered using a Visual Informational mode of delivery (ie, a video demonstrating the new process) or a group-based mode of delivery, which might use a weekly meeting to discuss the new process."				
			Sys Review: St Quinton, T., Morris, B., Pickering, D. et al. Behavior Change Techniques and Delivery Modes in Interventions Targeting Adolescent Gambling: A Systematic Review. J Gambl Stud 38, 1503–1528 (2022). https://doi-org.libproxy.ucl.ac.uk/10.1007/s10899-022-10108-8	Results: "The reviewed studies contained a total of six MODs: face-to-face; website; computer; playable electronic storage (i.e., video tapes, DVDs); printed publication; and video game (see Table 5)..."				
			Sys Review Protocol: Kenny E, McEvoy JW, McSharry J, Collins LM, Taylor RS, Byrne M. Are behaviour change techniques and intervention features associated with effectiveness of digital cardiac rehabilitation programmes? A systematic review protocol. HRB Open Res. 2021;4:88. Published 2021 Aug 11. doi: 10.12688/hrbopenres.13355.1	Data extraction: Intervention: mode of delivery using the mode of delivery ontology v1				
			Interv development: Lucci, VE.M., McKay, R.C., McBride, C.B. et al. Barriers and facilitators to changing bowel care practices after spinal cord injury: a Theoretical Domains Framework approach. Spinal Cord 60, 664–673 (2022). https://doi.org/10.1038/s41393-021-00743-0	Results: "Human (61%), digital platforms (33%) and print material (6%) were identified as potential modes of delivery. It was unclear how human interaction was to be used as a mode of delivery with 82% (n = 9) of human modes of delivery coded as ‘unclear’. However, digital and print material included email (15%), websites (23%), instant messages (8%) and leaflets (8%)."				
**Intervention Setting**	30	5	Sys Review: Jackson, S., Brown, J., Norris, E., Livingstone-Banks, J., Hayes, E., & Lindson, N. (2022). Mindfulness for smoking cessation. *Cochrane Database of Systematic Reviews*, (4).	Text only within Characteristics of Included studies table: e.g. "Setting: community"	2	Sys Review protocol: Silva, C., Presseau, J., Dinsmore, J., van Allen, Z., & Marques, M. (2022). Protocol for two interrelated systematic reviews of multiple health behaviour change interventions in healthcare. PsyArXiv. https://psyarxiv.com/7dwrv/download?format=pdf	Upper levels only	https://osf.io/3cbmk
			Interv development: Umaefulam, V., Wilson, M., Boucher, M. C., Brent, M. H., Dogba, M. J., Drescher, O., ... & Presseau, J. (2023). The co-development of a linguistic and culturally tailored tele-retinopathy screening intervention for immigrants living with diabetes from China and African-Caribbean countries in Ottawa, Canada. BMC Health Services Research, 23(1), 1–19.	In Methods only: "We identified the modes and settings of delivering behaviour change interventions [37, 38], agreed on materials to create, prototypes, and how to integrate other barriers and effective strategies not identified in the co-development workshops"		Scoping review: Wuerstl, K. R., Todd, K., Lawrason, S., Shwed, A., Holmes, B., & Gainforth, H. L. (2023). Theoretical components of smoking cessation interventions for persons with physical disabilities: A scoping review. Addictive Behaviors, 107762.	Full ontology	https://osf.io/54mhb
			Scoping review: Wuerstl, K. R., Todd, K., Lawrason, S., Shwed, A., Holmes, B., & Gainforth, H. L. (2023). Theoretical components of smoking cessation interventions for persons with physical disabilities: A scoping review. Addictive Behaviors, 107762.	Results: "A total of 20 intervention settings (comprised of 12 unique intervention settings) were extracted from the included articles. Interventions used, on average, 2.2 intervention settings, ranging from zero to five..."				
			Intervention: McMahon, S. K., Macheledt, K., Choma, E. A., Lewis, B. A., Guan, W., Wyman, J. F., & Rothman, A. J. (2023). Rethinking how and when to report descriptions of behavior change content within interventions: a case study of an ongoing physical activity trial (ready steady 3.0). Translational Behavioral Medicine, 13(6), 368–379.	Method: "The setting of the intervention was in five urban neighborhoods near community centers accessible to older adults. "				
			Interv development: Osborne, C. & Norris, E. (2022) Pre-registration as behaviour: developing an evidence-based intervention specification to increase pre-registration uptake by researchers using the Behaviour Change Wheel, Cogent Psychology, 9:1, DOI: 10.1080/23311908.2022.2066304	Results: "..university facility (BCIO:026028).."				
**Intervention Source**	14	3	Sys Review: Jackson, S., Brown, J., Norris, E., Livingstone-Banks, J., Hayes, E., & Lindson, N. (2022). Mindfulness for smoking cessation. *Cochrane Database of Systematic Reviews*, (4).	Text only within Characteristics of Included studies table: e.g. "Type of therapist/provider: each session was co-led by 2 trained group leaders, including a physician, doctoral-level psychologists, and clinical psychology doctoral students"	2	Sys Review protocol: Silva, C., Presseau, J., Dinsmore, J., van Allen, Z., & Marques, M. (2022). Protocol for two interrelated systematic reviews of multiple health behaviour change interventions in healthcare. PsyArXiv. https://psyarxiv.com/7dwrv/download?format=pdf	Upper levels only	https://osf.io/3cbmk
			Scoping review: Wuerstl, K. R., Todd, K., Lawrason, S., Shwed, A., Holmes, B., & Gainforth, H. L. (2023). Theoretical components of smoking cessation interventions for persons with physical disabilities: A scoping review. Addictive Behaviors, 107762.	Results: "A total of 35 intervention sources (comprised of 15 unique intervention sources) were extracted from the included articles. There were 29 intervention sources extracted from the intervention arms and six from the control arms.."		Scoping review: Wuerstl, K. R., Todd, K., Lawrason, S., Shwed, A., Holmes, B., & Gainforth, H. L. (2023). Theoretical components of smoking cessation interventions for persons with physical disabilities: A scoping review. Addictive Behaviors, 107762.	Full ontology	https://osf.io/v7j3b
			Interv development: Osborne, C. & Norris, E. (2022) Pre-registration as behaviour: developing an evidence-based intervention specification to increase pre-registration uptake by researchers using the Behaviour Change Wheel, Cogent Psychology, 9:1, DOI: 10.1080/23311908.2022.2066304	Results: "Researcher (BCIO:010083); relatedness between person source and the target population (BCIO:010094); expertise of person source (BCIO:010120).."				
**Mechanisms of Action**	2	0			0			
**Behaviour Change Technique Ontology (BCTO)**	7	0			0			
**Style of Delivery**	0	0			0			

### ii) Development of data extraction template

A prototype BCIO data extraction template was developed by: a) identifying an appropriate software to host the template, based on ease-of-use and accessibility, and b) compiling the identified examples of BCIO annotations from published papers.

### iii) Piloting and revising the data extraction template

The prototype BCIO data extraction template was piloted to annotate papers using the BCIO in a systematic review of digital interventions to address children’s fruit and vegetable consumption ( Froome *et al.*, 2023). Papers in this systematic review were double-coded using the ontologies by two authors (HF & EN), and appropriate revisions made. Instructions on how to use the template were added. The revised template was reviewed by the study team to produce ‘BCIO data extraction template v1’.

### iv) Launch and maintenance of data extraction template

The BCIO data extraction template v1 was made available online on the Open Science Framework and promoted via social media. A feedback portal was established to enable users to suggest improvements for the template.

## Results

### i) Scoping review of papers citing the BCIO

BCIO annotations were identified in 14 papers; their details are shown in Table 1, with all 14 papers using the Mode of Delivery Ontology ( Encantado *et al.*, 2022; Giroux *et al.*, 2021; Jackson *et al.*, 2022; Keller *et al.*, 2023; Kenny *et al.*, 2021; Lucci *et al.*, 2022; McClatchey *et al.*, 2024; McMahon *et al.*, 2023; Osborne & Norris, 2022; Shwed *et al.*, 2023; Silva *et al.*, 2022; St Quinton *et al.*, 2022; Umaefulam *et al.*, 2023; Wuerstl *et al.*, 2023), six papers using the Intervention Setting Ontology ( Jackson *et al.*, 2022; McMahon *et al.*, 2023; Osborne & Norris, 2022; Silva *et al.*, 2022; Umaefulam *et al.*, 2023; Wuerstl *et al.*, 2023; and four papers using the Intervention Source Ontology ( Jackson *et al.*, 2022; Osborne & Norris, 2022; Silva *et al.*, 2022; Wuerstl *et al.*, 2023). No papers were identified using the Mechanisms of Action, BCT and Style of Delivery ontologies.

Supporting documentation further detailing BCIO annotations was identified in four papers using the Mode of Delivery Ontology ( Encantado *et al.*, 2022; Silva *et al.*, 2022; Umaefulam *et al.*, 2023; Wuerstl *et al.*, 2023), two papers using the Intervention Setting and Intervention Source ontologies ( Silva *et al.*, 2022; Wuerstl *et al.*, 2023). This supporting documentation was provided via Open Science Framework ( Silva *et al.*, 2022; Wuerstl *et al.*, 2023) or additional documentation within the paper ( Encantado *et al.*, 2022; Umaefulam *et al.*, 2023).

### ii) Development of the prototype data extraction template

Microsoft Excel was used, as a widely available and routinely used software, with formats (.csv, .xlsx) that can be uploaded within journal submission systems. The template had a wide structure, with each ontology entity presented widthwise and each ontology level presented heightwise ( Encantado *et al.*, 2022). Associated papers coding using each ontology were noted (Pilot 1 of BCIO data extraction template https://osf.io/c89fv).

### iii) Piloting and revising the data extraction template

Revisions were made after piloting the BCIO data extraction template in an ongoing systematic review ( Froome *et al.*, 2023). Definitions and unique IDs for ontology entities were added. The template structure was revised to more clearly reflect the structure of ontologies, with levels widthwise, reflecting the structure used by Wuerstl *et al.* (2023), and papers were presented in columns rather than rows. For each paper, separate columns were added to represent ‘entity present’: i.e the entity being present in the given paper, and ‘evidence’ for quotes reflecting entity presence to be pasted directly into the document. Initially, the presence of an entity was indicated by highlighting the cell (as in Wuerstl *et al.*, 2023). However, after external feedback and discussions with the team, it was decided that the presence of an entity should be indicated by a ‘1’ in the relevant cell. This is to enable it to be computer readable. Columns for intervention and comparator conditions were added, to enable both to be annotated. The template allows users to add subsequent columns for each study included in their evidence synthesis.

All published ontologies were added into the template, with a tab for each ontology: Mode of Delivery, Setting, Source, Mechanism of Action, BCT Ontology and Style of Delivery. An ‘Intro’ guidance tab was added as the first tab in the document to include information on what the template can be used for, what ontologies are included within it and links to the associated papers, where to provide feedback on the template (specified in the next step), and how to complete the template. This revised template was piloted which confirmed the structure was clearer and much improved ( Froome *et al.*, 2023) (Pilot 2 of BCIO data extraction template https://osf.io/ntbce).

### iv) Dissemination and maintenance of data extraction template

The BCIO data extraction template v1 was made available on Open Science Framework ( https://osf.io/x6afp) and as Extended Data, and its citation generated ( Norris *et al.*, 2023). A feedback portal was established on Google Forms ( https://docs.google.com/forms/d/e/1FAIpQLSexgqQ1flljAfCnuCd3QdKYy6Yze3IFJmjBCelySgjXDNsjgA/viewform) to enable suggestions from users for further improvement. Questions include: a) ‘Please let us know if you have any queries on how to use this template?’, b) ‘Please let us know how you think this template can be improved’ (both free-text responses), c) Have you developed and alternative template that you wish to share?’ (Yes/No; if ‘Yes’ then requested to send to paper’s lead author) and d) ‘If you wish, please provide us with your contact email’. The BCIO data extraction template v1 and feedback portal were promoted via the Human Behaviour-Change Project social media accounts (X and LinkedIn) and BCIO website ( https://www.bciontology.org/use-evidence-synthesis). The template was intentionally labelled ‘v1’ to allow for amendments based on user feedback, addition of new ontologies as they become published (see collection https://wellcomeopenresearch.org/collections/humanbehaviourchange), and updates to existing ontologies that have been published, since ontologies are dynamic and expected to change over time as they are refined (see OBO Foundry Principle 16).

## Discussion

This paper presents development of a data extraction template to support annotations using the BCIO. This tool currently features entities from the six component ontologies within the BCIO that are published: mode of delivery ( Marques *et al.*, 2020), setting ( Norris *et al.*, 2020), source ( Norris *et al.*, 2021), mechanisms of action ( Schenk *et al.*, 2023), behaviour change techniques ( Marques *et al.*, 2023), and style of delivery ( Wright *et al.*, 2023). More ontologies within the BCIO are underway and will be added into this template once developed: human behaviour, fidelity, schedule, dose, engagement, and population. ****Annotation guidance manuals**** specific to each ontology is available online ( https://drive.google.com/drive/folders/1cJ53d0hBxHdSHYjf55c2S7NuJ03kXMFe) to use alongside the template. These manuals provide information on how to annotate entities within each ontology, and advice and rules for making decisions.

Annotation of behaviour change intervention reports according to the BCIO is made simpler with this data extraction template. All entities within BCIO are presented in one document, available in the readily accessible and easy-to-use format of Microsoft Excel. This resource facilitates the annotation of intervention protocols and evaluation reports to allow cumulative understanding of the complex question central to changing human behaviour: *“When it comes to behaviour change interventions: What works, compared with what, for what behaviours, how well, for how long, with whom, in what setting, and why?”* ( Michie *et al.*, 2017). We encourage researchers and practitioners that use this template to share their annotations of intervention reports through an open access repository (e.g. Open Science Framework, which can generate a link), so that their work can be built upon, and potentially avoid duplication of effort.

## Strengths and limitations

This template provides an easy-to-use method of annotating according to the BCIO using widely available software (Microsoft Excel). It also allows users to view the ontologies within the BCIO in-full and in a hierarchical structure without using ontology-specific ****Web Ontology Language (OWL)**** format ( http://humanbehaviourchange.org/ontology/bcio.owl). The template has an ongoing route to improvement based on user suggestions, via the feedback portal. However, a limitation of using Microsoft Excel for this BCIO coding template is that updates to the ontologies within it require manual adjustments and maintenance, as opposed to being automatically integrated into the document.

Manual data extraction can be time-consuming, and a degree of training may be needed to understand and become familiar with the BCIO and its entities before undertaking the data extraction stage. A training programme has been developed and is available at https://www.bciontology.org/training. The training aims to help people use the BCIO effectively and encourage them to build the BCIO into their routine workflows. Developments such as natural language processing, machine learning and artificial intelligence provide new opportunities for automated data extraction, reducing the time necessary to complete or update an evidence synthesis ( Jonnalagadda *et al.*, 2015).

## Future research using this BCIO data extraction template

The BCIO data extraction template can be used to annotate the BCIO within evidence synthesis studies, as well as to describe new intervention studies. In the future, we will update this template based on feedback from users, additions of newly completed ontologies within the BCIO, and revisions to existing ontologies. When future versions of the template are released, we will change to v2, v3 etc. Users should specify exactly which version they have used in their work. The template URL ( https://osf.io/x6afp) will always link to the most current version, with previous version accessible by clicking on the Revisions tab on Open Science Framework.

## Consent

Any respondents to the Google Forms feedback portal are asked for their informed consent. Respondent indicate their consent by ticking a box.

## Figures and Tables

**Table A1.** Glossary of terms.

Term	Definition	Source
**Annotation**	Process of coding, or tagging, selected parts of documents or other resources to identify the presence of ontology entities.	Michie *et al.* (2017)
**Annotation guidance manual**	Written guidance on how to identify and tag pieces of text from intervention evaluation reports with specific codes relating to entities in the ontology, using for example EPPI-Reviewer software.	Michie *et al.* (2017)
**Class**	A category of entities as represented in an ontology.	Arp *et al.* (2015)
**Entity**	Anything that exists or can be imagined, including objects, processes, and their attributes. It Includes mental process, i.e., the process and content of cognitive representations, and emotions. Entities can be represented hierarchically by parent and child classes (see definition of **parent class**).	Arp *et al.* (2015)
**Interoperability**	Two systems are interoperable to the extent that the data in each system can be used by the other system. Note: An ontology is interoperable with another ontology if it can be used together with the other ontology.	http://www.obofoundry.org/principles/fp-010-collaboration.html
**Open Biological and Biomedical Ontology (OBO) Foundry**	A collective of ontology developers that are committed to collaboration and adherence to shared principles. The mission of the OBO Foundry is to develop a family of interoperable ontologies that are both logically well-formed and scientifically accurate.	Smith *et al.* (2007) www.obofoundry.org/
**Ontology**	A standardised representational framework providing a set of entities for the consistent description (or “annotation” or “tagging”) of data and information across disciplinary and research community boundaries.	Arp *et al.* (2015)
**Parent class**	A class within an ontology that is hierarchically related to one or more child classes (subclasses) such that all members of the child class are also members of the parent class, and all properties of the parent class are also properties of the child class.	Arp *et al.* (2015)
**Relation**	The manner in which two entities are connected or linked.	Arp *et al.* (2015)
**Uniform Resource Identifiers (URI)**	A string of characters that unambiguously identifies an ontology or an individual entity within an ontology. Having URI identifiers is one of the OBO Foundry principles.	http://www.obofoundry.org/principles/fp-003-uris.html
**Web Ontology Language (OWL)**	A formal language for describing ontologies. It provides methods to model entities of “things”, how they relate to each other and the properties they have. OWL is designed to be interpreted by computer programs and is extensively used in the Semantic Web where rich knowledge about web documents and the relations between them are represented using OWL syntax.	https://www.w3.org/TR/owl2-quick-reference/

## Data Availability

Open Science Framework: Human Behaviour-Change Project. https://doi.org/10.17605/OSF.IO/EFP4X ( West *et al.*, 2023b). This project contains the following extended data: Pilot 1 of BCIO data extraction template ( https://osf.io/c89fv) Pilot 2 of BCIO data extraction template ( https://osf.io/ntbce) BCIO data extraction template v1 ( https://osf.io/x6afp) Data are available under the terms of the Creative Commons Attribution 4.0 International license (CC-BY 4.0).
