# Supplementary material for: A data extraction template for the behaviour change intervention ontology
Source: Wellcome Open Res. 2024 Mar 26;9:168. [Version 1] doi: 10.12688/wellcomeopenres.20872.1 (PMC11170071; doi:10.12688/wellcomeopenres.20872.1)
Supplement: Peer review history

# Reviewer report: Reviewer response for version 1

**DOI:** 10.21956/wellcomeopenres.23094.r86252

**Published:** 2024-07-15

**Authors:** Tamla Evans

**Affiliations:** 1 Obesity Institute, Leeds Beckett University School of Health (Ringgold ID: 151650), Leeds, England, UK

Thank you for the opportunity to review this important study which addresses the gap in tools available to support use of the recently developed BCIO for the purposes of data extraction. Development of this tool will increase the accessibility and usability of the BCIO and facilitate the consistent reporting of behaviour change interventions needed for successful systematic reviews and meta-analyses.

 The manuscript provides a concise overview of the development process which included a scoping review and pilot test for version 1, with further developments planned for version 2 based on user feedback.

 I have provided a few comments below on how this manuscript could be further improved.

 Methods

i) Please describe the inclusion/exclusion criteria and screening process for these papers to enhance transparency and replicability. For example, Table 1 suggests that papers were included if they reported BCIO coding in-text?
iii) "The prototype BCIO data extraction template was piloted to annotate papers using the BCIO in a systematic review of digital interventions” – please reword as this could be mistaken as only papers using the BCIO were eligible for inclusion in the review cited.

  Results

i) No papers were identified using the Mechanisms of Action, BCT and Style of Delivery ontologies. Please can you describe the process of developing these sections of the prototype template in the absence of this information?
 iv) Please can you state the month and year in which the template was made available on OSF.
iv) Great to see the inclusion of a feedback portal here. It is important to note that the template authors are all familiar with the BCIO. Therefore, piloting of the template by study groups outside of this team is important to improve its usability across disciplines. Has any feedback been received yet? It would be useful to include this, although I appreciate not much time has lapsed since the OSF publication.

Discussion

 1. Strengths and limitations: In relation to my previous comment, I think it is important to acknowledge that the study authors are familiar with the BCIO and likely this would have influenced the perceived usability of the template. The template will be applicable to interventions across a range of behaviours and disciplines. There is often limited behavioural science expertise within research, policy, and intervention development teams, therefore the feedback will be vital for optimising the accessibility of the tool so that it can achieve its desired impact.

Is the work clearly and accurately presented and does it cite the current literature?

Yes

If applicable, is the statistical analysis and its interpretation appropriate?

Not applicable

Are all the source data underlying the results available to ensure full reproducibility?

Yes

Is the study design appropriate and is the work technically sound?

Yes

Are the conclusions drawn adequately supported by the results?

Partly

Are sufficient details of methods and analysis provided to allow replication by others?

Partly

Reviewer Expertise:

Health Psychology and Behavioural Science

I confirm that I have read this submission and believe that I have an appropriate level of expertise to confirm that it is of an acceptable scientific standard, however I have significant reservations, as outlined above.

# Reviewer report: Reviewer response for version 1

**DOI:** 10.21956/wellcomeopenres.23094.r82422

**Published:** 2024-07-12

**Authors:** Jacqueline L Mair

**Affiliations:** 1 Singapore-ETH Centre, Campus for Research Excellence And Technological Enterprise (CREATE), Singapore, Singapore; 2 Saw Swee Hock School of Public Health, National University of Singapore, Singapore, Singapore

This paper provides a very nice overview of the process undertaken to develop a data extraction tool that can be used to systematically and consistently describe behaviour change interventions via ontologies. With widespread uptake amongst the behavioural science community (and beyond) this will facilitate better reporting and better evidence synthesis to enhance our understanding of the effectiveness of behaviour change interventions.

 I have a few comments for consideration:

- When exactly was the BCIO published? I expect few published papers are citing the ontologies (yet), rather than the BCTTv1 for example. But my question is why the scoping review of citing papers step was needed to develop the template? Could you elaborate a little on why this was necessary and how it helped?
- Only one database (Google Scholar) has been used to search citing articles. While Google Scholar may have the largest coverage of the most common databases, it is possible that relevant articles may have not been included so you may wish to mention that.
- From my understanding, the data extraction template is primarily (at least for now) to be used when conducting systematic reviews of the literature. I have two thoughts about this: (1) I wonder why you choose to publish this template now rather than wait until all ontologies are included? Would this not lead to mixed/incomplete reporting? (2) How will this link to existing reporting guidelines which don’t necessarily ask data extraction templates to be published? Are there plans to update existing reporting checklist to ensure this step is completed?
- To answer questions related to “what works” wouldn’t the template need to also include data extraction of primary/secondary outcomes or some measure of effect? I didn’t see this mentioned so I am interested to know how you will do this, particularly with respect to predicting behavioural outcomes.
- Somewhat linked to the previous comment, I understand one of the motivations for the approach was to enable AI and ML to read and synthesis the data and make predictions on outcomes. Are excel documents the best way to do this? You hint towards this in the paper but it’s not entirely clear how this will be realised and I think it would be good to expand on this. If this is the first step in the process, what is the roadmap going forward?  
- Is it expected that authors will also use and publish the excel template when describing the development of interventions and how will that impact on the publication process in terms of reporting standards etc?
- I’m wondering whether you can provide some brief advice on the ‘how to use’ highlights? I appreciate there are detailed manuals, but this paper could highlight the key points for readers. For example, with mode of delivery, things can get complicated with digital interventions - how do you distinguish between mobile device BCIO:011012 and mobile app BCIO:011028 and even messaging BCIO:011024? In the case of a whatsapp intervention, should all three be coded? Some key considerations would be helpful in this manuscript. (I also noted on the Mode of Delivery manual that you suggest having a separate extraction sheet for each arm– why is that? Your template has the intervention and comparator together.)
- Minor point, but it’s not immediately clear what is meant by upper and lower levels until you look into the template itself. I wonder whether there is another way to describe this sort of hierarchical structure?
- Please provide dates of when all documents were made available.
- In the introduction, this sentence reads strangely “ The BCIO is an advance as it allows…”. Maybe include “…an advance of the BCTTv1…”
- Have any changes been made based on the request for feedback?

Is the work clearly and accurately presented and does it cite the current literature?

Yes

If applicable, is the statistical analysis and its interpretation appropriate?

Not applicable

Are all the source data underlying the results available to ensure full reproducibility?

Yes

Is the study design appropriate and is the work technically sound?

Yes

Are the conclusions drawn adequately supported by the results?

Yes

Are sufficient details of methods and analysis provided to allow replication by others?

Yes

Reviewer Expertise:

lifestyle behaviour change, mHealth, intervention development and evaluation

I confirm that I have read this submission and believe that I have an appropriate level of expertise to confirm that it is of an acceptable scientific standard.

# Reviewer report: Reviewer response for version 1

**DOI:** 10.21956/wellcomeopenres.23094.r86251

**Published:** 2024-06-18

**Authors:** Amy Rodger

**Affiliations:** 1 Edinburgh University, Edinburgh, UK

This paper describes the creation of the Behaviour Change Intervention Ontology (BCIO) data extraction template. The template aims to help researchers annotate evidence synthesis and intervention development studies using the BCIO. The authors clearly justify the purpose of this template and provide a detailed explanation of its development in the method section. Additionally, they offer easily accessible links to the template and supplementary material, allowing newcomers to the template and ontologies in general to engage with these tools.

 While I haven't utilized BCIO in my previous systematic reviews or intervention development work, I can appreciate the benefits these tools will offer researchers like myself in the future.

 I agree with the suggestions made by the previous reviewers. I also have one minor suggestion to enhance the reader's understanding of the role played by Froome et al. (2023) in the development of this template:

 In the introduction, the authors state: “For example, in annotating BCIO entities for a systematic review of digital interventions to address children’s fruit and vegetable consumption (Froome et al., 2023), it was apparent that there was a need for a single data extraction template covering all component ontologies within the BCIO.” It would be helpful to be given a specific reason why it was apparent this data extraction template was needed - especially since this paper was used to pilot the template during the development. Additionally, it would be helpful for the authors to briefly clarify the role the paper will play in the development of the template when it is introduced in the introduction.

Is the work clearly and accurately presented and does it cite the current literature?

Yes

If applicable, is the statistical analysis and its interpretation appropriate?

Not applicable

Are all the source data underlying the results available to ensure full reproducibility?

No source data required

Is the study design appropriate and is the work technically sound?

Yes

Are the conclusions drawn adequately supported by the results?

Yes

Are sufficient details of methods and analysis provided to allow replication by others?

Yes

Reviewer Expertise:

Developing and evaluating behaviour change interventions; Systematic reviews; No applied experience using the BCIO.

I confirm that I have read this submission and believe that I have an appropriate level of expertise to confirm that it is of an acceptable scientific standard.

# Reviewer report: Reviewer response for version 1

**DOI:** 10.21956/wellcomeopenres.23094.r86258

**Published:** 2024-06-12

**Authors:** Nikos Ntoumanis

**Affiliations:** 1 Danish Centre of Motivation and Behaviour Science (DRIVEN), University of Southern Denmark, Odense, Denmark

With the development of the new ontologies, a data extraction template that integrates all available ontologies is a welcome addition to the literature. The manuscript describes in a clear and concise way the steps used in the development of this template. Below I offer some minor thoughts and suggestions for the authors’ consideration.

1. The labeling of the template as “v1” could also be reflected in the title.
2. One thought I had reading this paper is that with the development of new ontologies, each of which will have its own guide for coding, using this template in an Excel format will become increasingly cumbersome. Surely, the way forward would be to develop a free software where all the ontologies and the user guide for each would be integrated in a user-friendly way? Something to perhaps consider mentioning in the Discussion.
3. In the abstract, it would be useful for the reader to know what ontologies are included in the template. Also, consider changing “facility” to “option”.
4. Introduction: In the second paragraph it is mentioned that ontologies follow principles but only one is mentioned (being openly available). Maybe the phrasing should be changed to “one principle the ontologies should follow is…” or something similar.
5. Method/Results: Can the authors report the inter-rater agreement in the double-coding they did in the piloting phases? I was also surprised to read the statement that “Definitions and unique IDs for ontology entities were added”. Which ontologies were altered and how exactly? Have the authors who developed these ontologies being involved in such changes? More information here would be useful, as this reads like a substantial change.

Is the work clearly and accurately presented and does it cite the current literature?

Yes

If applicable, is the statistical analysis and its interpretation appropriate?

Not applicable

Are all the source data underlying the results available to ensure full reproducibility?

Yes

Is the study design appropriate and is the work technically sound?

Yes

Are the conclusions drawn adequately supported by the results?

Yes

Are sufficient details of methods and analysis provided to allow replication by others?

Partly

Reviewer Expertise:

Motivation, health behaviour change, physical activity promotion

I confirm that I have read this submission and believe that I have an appropriate level of expertise to confirm that it is of an acceptable scientific standard.

# Reviewer report: Reviewer response for version 1

**DOI:** 10.21956/wellcomeopenres.23094.r80985

**Published:** 2024-05-24

**Authors:** Carolina C Silva

**Affiliations:** 1 Trinity College Dublin, Dublin, Ireland

The article describes the development of a data extraction template for annotating using existing ontologies within the Behaviour Change Intervention Ontology. The various steps involved in the development of this template are outlined, as well as a process to continuously update it.

 Having used some of these ontologies when conducting systematic reviews, I believe the development of this data extraction template will be very useful for assisting with annotation in evidence syntheses and for the reporting of intervention studies.

 Overall, the article is well-structured, clearly written, and makes a valuable contribution to the field of behaviour change. The suggested corrections below are minor and aim to improve its clarity and readability.

 Abstract:

 The phrase in the results section “The ‘BCIO data extraction template v1’ was produced following piloting and revision **, **incorporating a facility for user feedback.”, could be clearer. It would be helpful to specify what is meant by “incorporating a facility for user feedback”.

 Methods:

 In the section ‘iii) Piloting and revising the data extraction template’, I suggest adding the word ‘were’ in the sentence: “Papers in this systematic review were double-coded using the ontologies by two authors (HF & EN), and appropriate revisions made”.  The revised sentence should read: “… and appropriate revisions were made”.

 Results:

 There is a typo in section ‘iv) Dissemination and maintenance of data extraction template’, “c) Have you developed and alternative template that you wish to share?”. The word "and" should be replaced by "an".

 Discussion:

 I suggest clarifying earlier in the article that users should specify which version they have used in their work.

 Consent:

 There is a typo in the sentence ‘Any respondents to the Google Forms feedback portal are asked for their informed consent. Respondent indicate their consent by ticking a box.’ The word "respondent" should be pluralised to "respondents".

Is the work clearly and accurately presented and does it cite the current literature?

Yes

If applicable, is the statistical analysis and its interpretation appropriate?

Not applicable

Are all the source data underlying the results available to ensure full reproducibility?

Yes

Is the study design appropriate and is the work technically sound?

Yes

Are the conclusions drawn adequately supported by the results?

Yes

Are sufficient details of methods and analysis provided to allow replication by others?

Yes

Reviewer Expertise:

behaviour change, health psychology

I confirm that I have read this submission and believe that I have an appropriate level of expertise to confirm that it is of an acceptable scientific standard.
